# The Association and Prognostic Implications of Long Non-Coding RNAs in Major Psychiatric Disorders, Alzheimer’s Diseases and Parkinson’s Diseases: A Systematic Review

**DOI:** 10.3390/ijms252010995

**Published:** 2024-10-12

**Authors:** Lin Zhu, Meng Guo, Ke Li, Chuang Guo, Kuanjun He

**Affiliations:** 1 College of Life Sciences and Food Engineering, Inner Mongolia Minzu University, Tongliao 028000, China; a2278250569@163.com (L.Z.); lk081821@163.com (K.L.); guochuang@imun.edu.cn (C.G.); 2 Finance Office, Inner Mongolia Minzu University, Tongliao 028000, China; dameng@imun.edu.cn

**Keywords:** lncRNA, psychiatric disorders, neurodegenerative diseases, pathogenesis, biomarkers

## Abstract

The prevalence of psychiatric disorders and neurodegenerative diseases is steadily increasing, placing a significant burden on both society and individuals. Given the intricate and multifaceted nature of these diseases, the precise underlying mechanisms remain elusive. Consequently, there is an increasing imperative to investigate the mechanisms, identify specific target sites for effective treatment, and provide for accurate diagnosis of patients with these diseases. Numerous studies have revealed significant alterations in the expression of long non-coding RNAs (lncRNAs) in psychiatric disorders and neurodegenerative diseases, suggesting their potential to increase the probability of these diseases. Moreover, these findings propose that lncRNAs could be used as highly valuable biomarkers in diagnosing and treating these diseases, thereby offering novel insights for future clinical interventions. The review presents a comprehensive summary of the origin, biological functions, and action mechanisms of lncRNAs, while exploring their implications in the pathogenesis of psychiatric disorders and neurodegenerative diseases and their potential utility as biomarkers.

## 1. Introduction

Psychiatric disorders and neurodegenerative diseases are globally prevalent diseases with significant impact, causing long-term disability and imposing substantial economic and medical burdens on both families and societies [1,2,3]. Moreover, individuals suffering from these diseases often experience a range of comorbidities [1,2,3]. Because of the similarity of clinical symptoms among patients with different psychiatric disorders and neurodegenerative diseases, the accurate diagnosis of these diseases often presents challenges in drawing definitive conclusions [4]. The diagnostic criteria for psychiatric disorders currently depend on the International Classification of Diseases (ICD) and the Diagnostic and Statistical Manual of Mental Disorders (DSM) guidelines. These two diagnostic criteria, devoid of the necessity of assessing the patient’s biochemical markers, yield disparate conclusions among physicians based on their subjective expertise, and consequently contribute to a substantial misdiagnosis rate [5]. With the development of high-throughput technology, the diagnosis of these diseases at the molecular level has gradually become a possibility [5].

**Figure 1:**
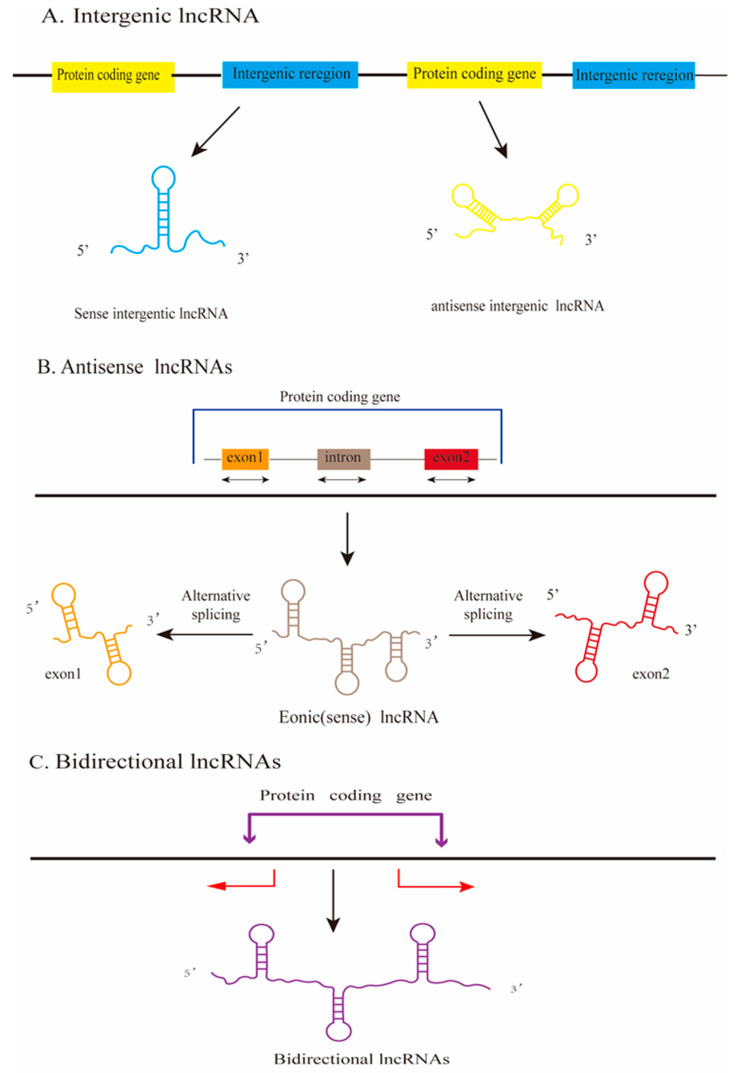
Processes of lncRNA production. (**A**) LncRNAs produced from non-coding regions between protein-coding genes are called intergenic lncRNAs; (**B**) lncRNAs originating from the exons of the protein coding segment of genes produce exonic lncRNAs; (**C**) bidirectional lncRNAs begin transcription within 1000 base pairs of the gene start site in the opposite direction.

Long non-coding RNAs (LncRNAs) are non-coding RNA molecules characterized by a length exceeding 200 nucleotides (nt) [6,7,8,9]. LncRNAs are produced in a similar manner to mRNAs, but their lower abundance and lack of open reading frames may result in the inability of lncRNAs to encode proteins [10]. Based on how lncRNAs are formed, they can be categorized into the following types: intronic RNAs, positive-sense RNAs, antisense RNAs, and bidirectional RNAs (see Figure 1) [6].

**Figure 2:**
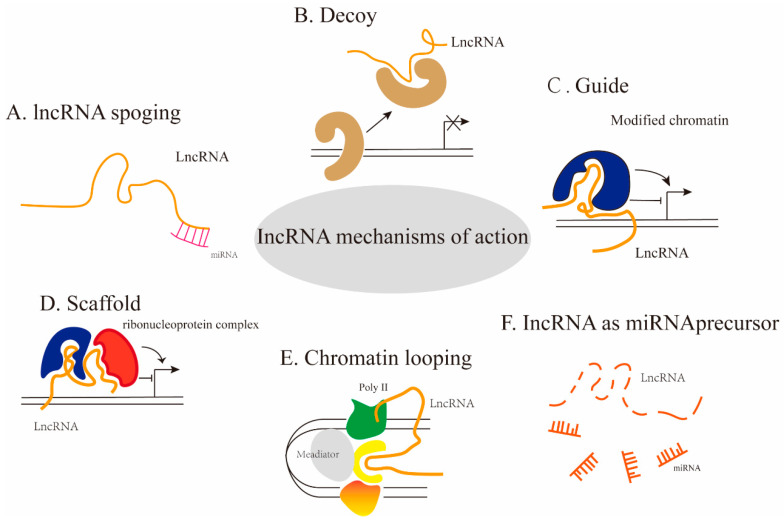
Action mechanisms of lncRNAs. (**A**). LncRNAs can act as “sponges” to affect miRNAs, promoting mRNA’s translation into protein; (**B**). LncRNAs can act as decoys to remove bound proteins; (**C**). LncRNAs can also act as guides to recruit proteins into the DNA; (**D**). LncRNAs can act as scaffolds to bring two or more proteins into complexes or spatial proximity; (**E**). LncRNAs can, by chromatin looping, promote mediator bind Poly II facilitate chromatin to loop; (**F**). some lncRNAs act as miRNA precursors by hydrolyzing.

With the in-depth study of lncRNAs, it has been shown that lncRNAs can act as ceRNAs, regulating the biological functions of miRNAs, such as acting as “sponges” to influence miRNAs [11]. LncRNAs can also act as scaffolds, decoys and guides [12,13]. LncRNAs can serve as decoys to regulatory proteins in order to enter and bind to DNA. LncRNAs can serve as junctions to bring proteins into the complex [14]. The localization of specific proteins to the correct site necessitates the involvement of certain lncRNAs, such as those implicated in stoichiometric compensation and imprinting, which function as guiding factors for silencing target genes. The action mechanisms of lncRNAs can be seen in Figure 2. Ample evidence suggests that lncRNAs exert crucial cellular functions, participate in gene regulation, modulate mRNA activities across various stages, and impact signal pathways [15], and lncRNAs with specific functions have the potential to be used as diagnostic markers for diseases [16]. LncRNAs are expressed at higher levels in the brain, and they play a key role in the regulation of brain-related functions [17]. Altered lncRNA integrity has been found to be associated with many diseases, and there is also evidence that abnormal lncRNAs contribute to the generation and development of psychiatric disorders and neurodegenerative diseases [3,18,19].

## 2. Method

### 2.1. Search Strategy

This review was conducted according to the Preferred Reporting Items for Systematic Reviews and Meta-Analyses (PRISMA) methodology [20]. We conducted a comprehensive search for published research articles and those on Google Scholar between the years 2005 and 2024 using the PubMed database. The most recent search was conducted on 12 July 2024. The title and abstract of each article were thoroughly screened by at least two authors for eligibility for full-text review. Only studies not definitively excluded by title and abstract were further evaluated. Each full-text article that met the requirements was then evaluated by three additional individuals to determine its inclusion in the study. This piece of systematic review was registered through INPLASY under registration number INPLASY202490076 and received DOI number 10.37766/inplasy2024.9.0076.

Inclusion criteria:

- English articles.
- Articles containing keywords related to lncRNA, biomarkers, psychiatric disorders, Alzheimer, Parkinson’s.
- Articles with abstracts.

Exclusion criteria:

- Articles with incomplete or unavailable full text.
- Articles written in languages other than English.
- Articles with low relevance to the scope of this review.

### 2.2. Data Items

Polymerization data extracted included analysis method, sequencing method, biomarker data information, association with lncRNA or not, description of results.

### 2.3. Risk of Bias and Quality Assessment

For the systematic evaluation of study quality and risk of bias, our review integrated qualitative and quantitative methods. The quality of the included studies was assessed using the Newcastle–Ottawa Scale [21]. To ensure objectivity and reproducibility, two researchers independently evaluated the studies, resolving any discrepancies through discussion. If consensus was unattainable, a third researcher resolved the differences.

## 3. Results

**Figure 3:**
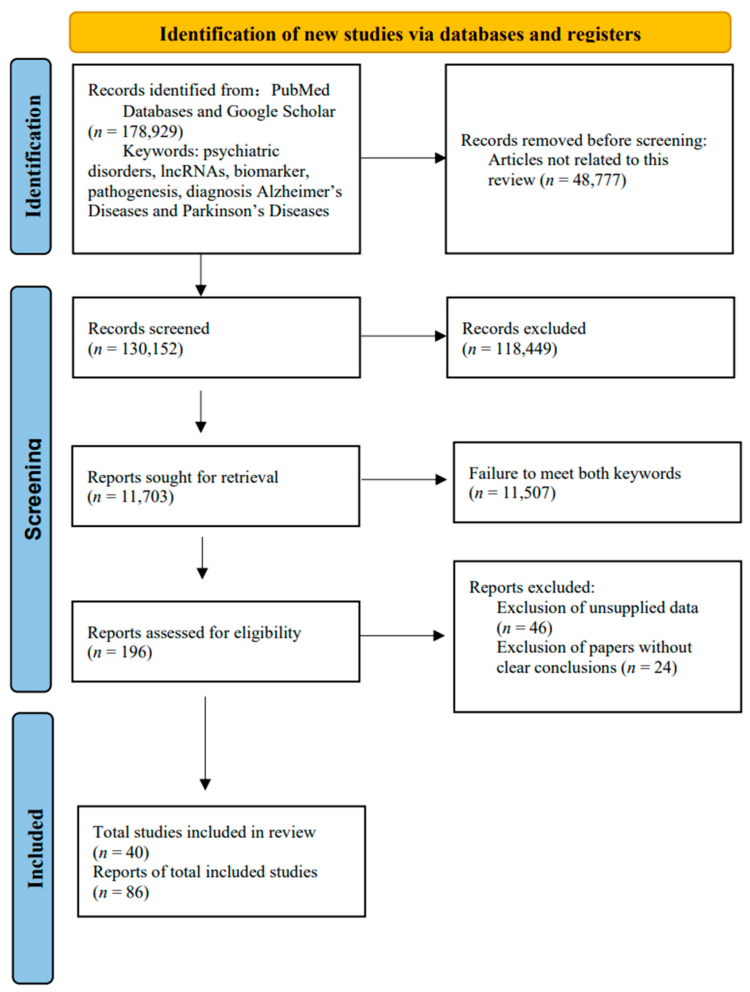
PRISMA flowchart.

All of the studies included in this review explored the value of lncRNA as the most important biomarker for psychiatric disorders, Parkinson’s disease (PD) and Alzheimer’s disease (AD). Initially, a comprehensive search yielded a total of 178,929 articles related to this review. After deleting duplicate articles, the remaining articles were screened based on title and abstract. Subsequently, a comprehensive full-text analysis of 196 articles was performed and included in this study. Among these 196 articles, the comparison between patients and healthy controls and the analysis of subjects’ work and differential expression revealed that differentially expressed lncRNAs were significantly associated with the development of these diseases, and the receiver operating characteristic (ROC) analysis showed that lncRNAs have a high diagnostic value for psychosis, as well as for AD and PD. Figure 3 illustrates the process of article screening using a PRISMA flowchart.

### 3.1. LncRNAs and Psychiatric Disorders

Psychiatric disorders mainly include schizophrenia (SCZ), major depressive disorder (MDD), and bipolar disorder (BD) [4]. Currently, it is widely recognized that psychiatric disorders are caused by complex interactions between genetic and non-genetic environmental factors [22]. Based on high-throughput sequencing technology, several aberrantly expressed lncRNAs have been found to be linked to psychiatric disorders, such as SCZ [23], MDD [24], BD [25], etc. The relationships between lncRNAs and psychiatric disorders can be seen in Table 1.

#### 3.1.1. LncRNAs and SCZ

SCZ is a psychiatric disorder with wide-ranging effects, which can be debilitating, affecting the health of the patients, causing mental deterioration and neurocognitive deficits [36,37,38,39]. However, the pathogenesis of SCZ is not yet clear. Researchers have found that the onset and progression of SCZ are linked with epigenetics, genetics and environment [40].

Much research has evidenced that lncRNAs participate in psychiatric disorders. Thus, researchers pay more attention to which lncRNAs have an important effect on these disorders. Gomafu, also known as MIAT (Myocardial infarction associated transcript), is found to interact with psychiatric disorders through lncRNA–miRNA interactions [30], and it has been found that reduced levels of Gomafu expression can be seen in the brains of postmortem schizophrenic patients [41]. In this study, Guo et al. (2022) analyzed the expression levels of lncRNA RP5-998N21.4 in peripheral blood samples using real-time quantitative PCR (RT–qPCR) obtained from 51 patients diagnosed with SCZ and 48 healthy controls [42]. Among the findings, significantly increased expression levels of lncRNA RP5-998N21.4 were found in individuals affected by SCZ [42]. By analyzing the RNA-seq data from the brain of monozygotic twins discordant for schizophrenia, the expression of lncRNA RP5-998N21.4 was also found to be significantly higher in the SCZ patients [42]. Gamma-Aminobutyric Acid (GABA) is associated with neuronal differentiation, and abnormal inhibitory function of GABA leads to SCZ, whereas lncRNA Evf2 regulates GABA and maintains the normal physiology of the organism [43]. According to Meng et al. (2018), the expression level of DiGeorge syndrome critical region gene 5 (DGCR5) was significantly reduced in brain tissue samples from deceased schizophrenic patients [44]. The expression of lncRNAs was analyzed in peripheral blood mononuclear cells (PBMCs) from SCZ patients and healthy individuals by qPCR [29,45]. It was found that the expression of multiple lncRNAs underwent differential changes [45,46]. In addition, researchers found that the types of lncRNAs changed in SCZ patients differed by gender [5,47]. For example, PVT1, FAS-AS1 and TUG1 showed greater differential changes in expression in males, while GAS5, NEAT1, THRIL and OIP5-AS1 were more differentially expressed in females [5,47]. NEAT1 affects oligodendrocyte dysfunction and myelin defects and, in the peripheral blood and cerebral cortex of patients with SCZ, their expression levels show a down-regulation [28,48,49].

The expression of crystallin Beta B1 (Crybb1) was inhibited in the cerebral cortex of mice that had knocked out the Gomafu gene [50]. The interaction of Gomafu with poly-comb repressive complex 1 (PRC1) in the promoter region of Crybb1 can be blocked, which leads to mice exhibiting anxious and disturbed behavior [51]. Researchers also found that the expression level of lncRNA NON-HSAT089444 was significantly up-regulated in PBMCs of SCZ patients. Knockdown of lncRNA NON-HSAT089444 decreased dopamine receptors’ (DR) D3 and DRD5 expression [46]. Conversely, overexpression of the lncRNA NON-HSAT089444 increased the expression levels of DRD3 and DRD5 [46].

In recent years, single-nucleotide polymorphisms (SNPs) in genes coding for lncRNAs have been associated with the pathogenesis of SCZ [51]. Rao et al. (2015) conducted a study that included two different phases of association analysis and found that rs1894720 of Miat was significantly associated with paranoid SCZ [52]. The study also found that reduced levels of lncRNA00461 expression in the hippocampus may be associated with the development of SCZ and other psychiatric disorders [53,54].

#### 3.1.2. LncRNAs and MDD

MDD is a leading contributor to morbidity and disability, affecting approximately 300 million individuals worldwide with a lifetime prevalence of around 16–17% in the general population [55,56]. Currently, there is uncertainty about the pathogenesis of MDD, but it is widely recognized to be related to genetic, environmental, social and developmental vulnerability and recovery factors [31,57,58].

Recently, the aberrant expression lncRNAs in the neural network of the brain and MDD patients’ peripheral blood were found by analyzing several high-throughput expression profiles [59]. In PBMC from patients suffering from depression, increased expression levels of 1556 lncRNAs were detected, along with decreased expression levels of 441 lncRNAs [60]. Researchers found that the expression of six lncRNAs (LINC02151, LNC02152, NONHSAG045500, LNC02153, NONHSAT034045, and NONHSAT142707). was significantly down-regulated in PBMCs of MDD patients [27]. These six lncRNAs were found to be present only in the PBMCs of MDD patients with a history of suicide attempts [27]. These findings suggest that these six lncRNAs have the potential to become useful biomarkers for suicidal ideation in MDD [61]. Issler et al. (2020) performed a study on brain tissues of MDD patients and found that lncRNAs were associated with differences in sex in MDD [62]. Additionally, the effects of lncRNA LNC00473 on the prefrontal cortex in females were found through primate experiments, and LNC00473 was found to alter neurophysiology and neuroendocrine effects through a chronic mouse stress model [62]. Combined with experiments on neuroblastoma cells of women, LNC00473 was found to bind primarily to genes that exert brain-related functions, some of which have been recognized with depression and anxiety [62]. Brain-derived neurotrophic factor (BDNF) is a crucial class of neurotrophic factors associated with the pathogenesis of MDD. In a murine model of chronic unpredictable mild stress (CUMS), reduced levels of lncRNA MIR155HG and BDNF were found in the hippocampus [63].

The results showed that lncRNA played a positive role in protecting CUMS mice by regulating the gene MIR155HG downstream of the miR-155/BDNF axis [63]. Serotonin transporter protein (SERT) plays a crucial role in synaptic clearance of 5-hydroxytryptophan (5-HTP) and serves as a potential target for SSRI-based anti-MDD therapies to inhibit the entry of 5-HTP. Conversely, overexpression of NONHSAG045500 led to a decrease in SERT in SK-N-SH neuroblastoma cells, which in turn compromised the efficacy of anti-multiple sclerosis medications and ultimately led to the failure of multiple sclerosis treatment [64]. Based on the results of qPCR analysis of blood samples from perinatally depressed patients, we found that the presence of six lncRNAs (NONSUSG010267, NONHSAT140386, NONHSAG004550, NONHSAT125420, NONHSAG013606 and NONMMUG014361) showed down-regulation [26]. After treatment, the expression of NONHSAG004550 and NONHSAT125420 showed significant up-regulation and these findings suggest that the combination of NONHSAG004550 and NONHSAT125420 is expected to be a new potential biomarker for MDD diagnosis [26].

#### 3.1.3. LncRNAs and BD

BD is a multifactorial condition that leads to severe chronic psychiatric disorders. It is characterized by alternating episodes of mania or hypomania and depression [65,66]. The prevalence of BD exceeds 1% in the global population and is characterized by an early onset. Moreover, BD is associated with premature mortality due to comorbidity with other disorders and an increased risk of attempted suicide. The onset of BD is influenced by a combination of genetic and environmental factors, with the estimated prevalence rate ranging from 60% to 85% [67,68,69]. Genome-wide association studies (GWAS) have identified several loci associated with BD. However, the etiology of BD and the underlying biological networks involved in the disease still largely elude our understanding.

LncRNAs as mediators of inflammatory regulation influence the brain and induce psychiatric disorders. COX-2 and NF-κB are key mediators in the inflammatory pathway, while PACER and NKILA are lncRNAs that regulate the expression of COX-2 and NF-κB. qPCR assay revealed that the expression levels of NKILA and COX2 were significantly decreased in patients with BD [32]. The analysis of ROC curves found that the NKILA could be used as a candidate biomarker for BD [32]. Hu et al. (2016) found that lncRNAs (PTCSC3, DISC2, XIST, and FTX) had characteristics such as specificity to brain regions and were predominantly enriched in pathways related to immune system development and oligodendrocyte differentiation. The abnormal expression of these lncRNAs was linked to DNA methylation. The association was identified by analyzing the brain transcriptomes of deceased patients with SCZ and BD [70]. Abnormalities in NF-κB lead to the development of MDD, which can be regulated by related lncRNAs, such as DICER1-AS1, DILC, and CHAST [35]. ROC curve analysis revealed that the AUC of CHAST was 0.83, which means that it has the potential to serve as a biomarker [35,66]. Through qPCR assay, the three lncRNAs (RMRP, CTC-487M23.5 and DGCR5) were detected in 50 BD patients and 50 healthy groups [33]. The expression levels of RMRP and CTC-487M23.5 were significantly up-regulated in the BD patients, and the level of expression DGCR5 did not have any significant change [33]. The ROC curves for RMRP and CTC-487M23.5 were 0.80 and 0.61, respectively. Furthermore, there was no correlation between the expression levels of RMRP and CTC-487M23.5 and other clinical features [33]. Downregulation of IFNG-AS1 was found to be accompanied by a decrease in IL-1B expression in the peripheral blood of the normal group versus BD patients [71]. Expression levels of lncRNA-p21, lncRNA-ROR and lncRNA-PINT in PBMCs were analyzed [34]. Significant alterations were observed in the expression levels of all three types of lncRNAs, which exhibited a strong positive correlation [34]. Furthermore, after conducting ROC curve analysis, the AUC values for these three types of lncRNAs exceeded 0.5 [34]. The different expression levels of these three lncRNAs in peripheral blood may serve as biomarkers for the diagnosis of patients with BD [34].

#### 3.1.4. LncRNA Expression Effects by Drug Treatment

In some studies, the expression of lncRNAs in patients undergoing antipsychotic treatment for SCZ and BD were analyzed [66,72]. The research revealed that MEG3 expression was significantly reduced in psychiatric participants taking antipsychotic medications compared to those who were medication-naive [73]. Additionally, no significant differences were observed in the expression levels of the lncRNAs PINT and GAS5 between the two groups [66]. One research conducted a comparison between patients receiving risperidone alone and a control group, and the results demonstrated that MEG3 expression was markedly lower in participants treated with risperidone compared to those who were not receiving the medication, while the expression levels of GAS5 and PINT remained unchanged between the two groups [73]. Notably, in SCZ patients receiving pharmacological treatment, the expression levels of lncRNAs Neat and MIAT returned to normal after therapy, contrasting with the decreased levels observed when the illness was left untreated [28].

### 3.2. LncRNA and Neurodegenerative Diseases

Neurodegenerative diseases include AD and PD, etc. Similarly, aberrantly expressed lncRNAs were observed in patients with neurodegenerative diseases, including PD and AD [74], etc. The relationships between lncRNAs and psychiatric disorders can be seen in Table 2.

#### 3.2.1. LncRNAs and AD

AD is a prevalent form of dementia, a relentless neurodegenerative condition marked by a profound deterioration in cognitive functions, leading to a significant disruption in the performance of everyday activities [81]. AD is clinically characterized by extracellular β deposition of Aβ peptide fibers and intracellular neurogenic fibers in tangles. The etiology of the disease is complex and not yet fully understood. In the observation of patients with different degrees of AD, the clinical features of the disease include neuroinflammation and neuronal and synaptic loss caused by microglia and astrocytes [81,82,83].

Most AD cases are categorized as sporadic late-onset AD [84]. AD research to date has focused on protein variants, mutations, and diversity, and the mechanisms by which they lead to AD production [85]. Several lncRNAs were abnormally expressed in the brain of AD in comparison with the healthy group, shedding light on the strong correlation between aberrant lncRNA expression and the onset and progression of AD pathology, highlighting the pivotal role of lncRNA dysregulation in the pathophysiology of this devastating neurodegenerative disorder, and these abnormally expressed lncRNAs were found to be associated with AD-related pathways, including Aβ and Tau production, clearance, and autophagy [86]. AD-associated lncRNAs have been found to be associated with synaptic and neuronal depletion, mitochondrial damage, and DNA damage [87].

Recently, it was found that the expression level of the lncRNA MAPT-AS1 is negatively correlated with the expression of protein of Tau, and MAPT-AS1 exerts a critical regulatory influence on the translation of Tau proteins by engaging in competitive ribosome binding at the ribosome entry site in the mRNA of MAPT, revealing a previously unrecognized mechanism for the complex process of lncRNAs in Alzheimer’s disease and other neurodegenerative diseases [76]. Similarly, Faghihi et al. (2008) found that lncRNA BACE1-AS is highly abundant in the neural network of the brain and encodes the β-secretase BACE1, which is involved in the process of amyloid synthesis and biochemical reactions as an enzyme synthesizing Aβ peptide [75]. BACE1 is highly expressed in the brain, and both its expression level and enzyme activity are markedly elevated in the AD brain. Obviously, the abnormal secretion of BACE1 causes amyloid to be precipitated, which affects the brain function, leading to AD disease [75]. LncRNAs BC200, 17A, NDM29, and 51A have been found to significantly elevate the production of Aβ, unveiling a crucial link between the abnormal expression of these lncRNAs and the observed pathology in the brains of patients afflicted with AD [88]. lncRNA 17A affects the precise splicing of the GABA B2 receptor, thereby hindering its intracellular signaling. Furthermore, under the influence of inflammatory stimuli, this lncRNA enhances Aβ secretion in neuroblastoma cells, thus identifying a molecular pathway that may contribute to the complex interaction between inflammation and Alzheimer’s disease pathology.

Synaptic deficits are observed as one of the clinical phenomena in patients with AD. Importantly, aberrant expression of BC200 hinders synapse formation at the translational level [88]. In neuroblastoma cells, the presence of 17A leads to a dramatic increase in amyloid β peptide (Aβ) secretion. This process ultimately leads to the production of Aβ, revealing a key link in the pathogenesis of Alzheimer’s disease [89]. Up-regulation of lncRNA NDM29 increases amyloid precursor protein synthesis, elevates the Aβ-42/Aβ-40 ratio, and increases the amount of Aβ secreted, inducing AD [90]. LncRNA-51A and SORL1 can affect β-amyloid formation through antisense overlaps and were found to have aberrant expression in the brain, contributing to AD disease [89]. A comprehensive study revealed aberrant expression in lncRNA expression profiles through microarray analysis of PBMCs obtained from blood samples from Alzheimer’s disease patients and healthy controls. The results showed that 14 lncRNAs exhibited up-regulation and 20 lncRNAs exhibited down-regulation in samples from AD patients, a discrepancy that reveals the intricate molecular structure behind the debilitating neurodegenerative disease [90]. RP3-522J7, miR3180-2 and miR3180-3 were found to be the highest co-expressed lncRNAs associated with AD by transcriptomic analysis [77].

#### 3.2.2. LncRNAs and PD

PD stands as the second most prevalent degenerative neurological disorder on a global scale, impacting approximately 2% of individuals aged 70 and above across the diverse tapestry of the world’s population [91,92]. PD is a complex neurological debilitating condition with an extremely complex pathophysiology that is characterized by selective degeneration of dopaminergic neurons in the substantia nigra, leading to the blockage of the nigrostriatal pathway, resulting in symptoms such as bradykinesia and gait abnormalities [91,93]. Neuronal damage is commonly observed in PD, and patients with PD usually have a persistent inflammatory response and increased neuronal damage [91,93,94]. The pathogenesis of PD remains unknown [95].

LncRNAs H19, GAS5, HAR1B and LNC01783 were detected as highly expressed in the brain of PD patients by qPCR analysis. There was a significant negative correlation between GAS5 expression levels and HY and Unified PD Rating Scale (UPDRS) scores. Such studies reveal a potentially important link between dysregulation of GAS5 and clinical severity of Parkinson’s disease [78]. By establishing a PD model, researchers found that MIAT may be involved in the pathogenesis of this neurodegenerative disease at both the molecular and cellular levels, bringing compelling evidence [96]. This also promotes inhibition of inflammasome activation and inhibits ROS generation [96]. Through the utilization of qPCR, an in-depth analysis of MIAT expression levels in PD mice across multiple brain regions yielded intriguing findings [97]. It was observed that MIAT exhibited varying degrees of down-regulation in the hippocampus, substantia nigra, striatum, and the entire brain. Furthermore, in vitro experiments conducted on SH-SY5Y cells revealed the remarkable potential of MIAT in safeguarding neurons, accentuating its role as a neuroprotective factor in the context of PD [97]. Up-regulation of lncRNA NEAT1 was found in a 1-methyl-4-phenyl-1, 2, 3, 6-tetrahydropyridine (MPTP) induced mouse model of PD [94]. It was shown that increased NEAT1 expression intricately stabilizes PINK1 expression, thus revealing a novel molecular mechanism with profound implications for the pathophysiology of PD [94].

The study unveiled that the overexpression of H19 in the induced mice model of PD suppresses miR-301b-3p, resulting in the up-regulation of the Wnt/β-catenin pathway via HRPT modulation, thereby ameliorating the loss of dopamine neurons [98]. Up-regulation of lncRNA HOTAIR increases ROS production and neuroinflammation, promoting PD [94]. PD mice induced by MPTP, lncRNA TUG1 and IL-6 and TNF-α were up-regulated [96]. At the same time; lncRNA UCA1 could affect the expression level of IL-6 and, IL-1β; up-regulation of lncRNA MALAT1 elevated pro-inflammatory factor expression, such as the binding of MALAT1 to EZH2 to regulate Nf2 in BV2 cells, and decreased the expression of Nf2, which can increase the probability of ROS and neuroinflammation [96,99].

The lncRNAs, SNCA-AS1, MAPT-AS1, AK127687 and AX747125, showed a significant tendency to be enriched in dopaminergic neurons and were detected in both peripheral and central tissues. The association of these lncRNAs with the pathogenesis of PD highlights their potential significance in unraveling the complex molecular basis of this debilitating neurodegenerative disease. Fan et al. (2019) also found that four differentially expressed level of lncRNAs (AC131056.3-001, HOTAIRM1, lnc-MOK-6:1 and RF01976.1-201) were up-regulated in leukocytes from patients of PD, and dysregulation of lncRNAs (AC131056.3-001 and HOTAIRM1) could lead to apoptosis of dopamine neurons [79]. Zou et al. (2020) used a microbead-based approach to study PD patients versus a healthy group and found that the expression of the protein encoded by lncRNA POU3F3 was highly up-regulated in PD [100]. Reduced levels of BDNF are thought to be involved in the pathogenesis of PD. Four lncRNAs, BDNF-AS, MIR137HG, MIAT, and PNKY BDNF-AS, MIR137HG, MIAT, and PNKY which are associated with BDNF were found to be down-regulation during transcription [101]. Currently, the lncRNA H19 has been implicated in the progression of PD as it is down-regulated in neuroblastoma cells in a PD mouse model [102]. In vitro experiments found that H19 inhibits apoptosis in neuroblastoma cells treated with membrane-permeable peptide (MPP) by regulating miR-585-3p [102].

### 3.3. LncRNAs as Candidate Biomarkers for the Diagnosis of Major Psychiatric Disorders, AD and PD

#### 3.3.1. LncRNAs as Candidate Biomarkers for the Diagnosis of Major Psychiatric Disorders

A biomarker is a biochemical indicator of changes and potential changes in the structure or function of systems, organs, tissues, cells and sub-cells [103]. Therefore, the search for reliable biomarkers is crucial for the early diagnosis, treatment and rehabilitation of psychiatric disorders [104]. Abnormal lncRNA expression is now being used as a diagnostic tool and is beginning to be used in cancer. In the field of neurological disease research, lncRNAs have been widely recognized as biomarkers with great potential, and thus their precise detection has become an important research focus.

The advancement of computerized algorithms has significantly enhanced the precision of lncRNA-based biomarkers. In patients with AD, the expression level of lncRNA MEG3 was down-regulated. Upregulation of lncRNA MEG3 was found to alleviate memory loss, and inhibit neuronal damage and associated apoptosis in rat models suffering from AD. The action mechanism of lncRNA MEG3 is associated with neuroinflammatory responses, rendering it a potential biomarker for neuroinflammation and a prospective class of biomarkers for psychiatric disorders [105,106]. Recently, researchers have discovered that lncRNAs have emerged as a new target for the diagnosis and treatment of a variety of human diseases, especially neurological disorders [107,108]. The levels of lncRNA MALAT1 in 50 individuals with phasic affective disorders and 50 healthy controls were analyzed using RT–qPCR [109]. The results showed a significant decrease in the expression of MALAT1, suggesting its potential as a diagnostic biomarker [109]. The identification and validation of lncRNAs exhibiting aberrant expression exclusively in specific psychiatric disorders represents a novel category of biomarkers that enhances precision for disease diagnosis.

#### 3.3.2. LncRNAs as Candidate Biomarkers for AD and PD

Comparison of the expression of LncRNAs BACE-AS1, NEAT1, and GAS5 in the AD patients and HC groups all showed a significant increase in expression and a total sensitivity and specificity of 74% and 88% based on the ROC, and it was found that lncRNAs have a high level of discriminatory power when used as diagnostic biomarkers for AD [110]. NEAT1 and BC200 plasma levels differentiated between AD patients and healthy controls, respectively, with sensitivities of 72% and 60% and specificities of 84% and 91%, respectively. By computer simulation of RNA-seq data, it was found that the levels of lncRNA NEAT1 and BC200 expressed in plasma may serve as potential blood-based biomarkers for AD occurrence and progression [111].

It was found that lncRNA MALAT1 is highly expressed in brain tissue and lncRNA was found to precede the course of PD disease, therefore lncRNA MALAT1 could serve as a potential biomarker for PD [112,113]. A significant increase in XIST expression was found in sera from patients with different levels of PD assessed by the Hoehn–Yahr scale using RT–qPCR, and an ROC curve was analyzed, suggesting that XIST has the potential to be used as a biomarker to diagnose PD [114]. The expression of lncRNA MEG3 in plasma was found to be negatively correlated with the NMSS score and, by comparing the expression level of MEG3 in PD and HCs, it was found that the expression of lncRNA MEG3 was significantly lower, while the NMSS score of PD patients was higher than that of the normal group. Therefore, the evidence above suggests that lncRNA MEG3 has the potential to be used as a candidate biomarker for the diagnosis of PD [115]. Silencing NEAT1 was found to attenuate symptomatic manifestations in PD mice and NEAT1 modulated miR-124-induced damage in PD neurons, which highlights NEAT1′s potential as a biomarker [116].

The current study identified LncRNAs as potential biomarkers for the diagnosis of major psychiatric disorders, AD, and PD, as detailed in Table 3.

## 4. Discussion

The review highlights the significant roles of lncRNAs in major psychiatric disorders, AD, and PD. Firstly, lncRNAs demonstrate high sensitivity and specificity in diagnosing major psychiatric disorders, AD and PD. For instance, LncRNAs BACE-AS1, NEAT1, and GAS5 demonstrated a robust discriminatory capability as candidate diagnostic biomarkers for AD [110]; differentially expressed lncRNA, MALAT1 [112,113], XIST [114], MEG3 [115] have potential as candidate diagnostic biomarkers for PD. Furthermore, lncRNAs show promise in early disease detection, which is crucial for conditions such as AD and PD, where early intervention can significantly affect disease progression and patient outcomes. At the same time, lncRNA-based tests can often be performed using blood samples, providing a less intrusive and potentially more accessible alternative to costly functional imaging techniques, like MRI or PET scans. These aspects underscore the high diagnostic potential of lncRNAs in psychiatric disorders, AD, and PD. The potential of lncRNAs as biomarkers and therapeutic targets opens exciting avenues for future research. Developing sensitive and specific detection methods for detecting lncRNA biomarkers will be crucial for their clinical application. Additionally, exploring the therapeutic modulation of lncRNA–microRNA interactions holds promise for innovative treatment strategies. Investigating ceRNA networks and their role in disease modulation could lead to novel interventions aimed at restoring normal cellular function. These findings contribute to a paradigm shift in the understanding of the molecular biology of neuropsychiatric and neurodegenerative disorders. Highlighting the central role of lncRNAs will pave the way for a new class of molecular targets that could revolutionize diagnosis and treatment.

Research indicates that SNPs associated with psychiatric disorders may play a significant role in regulating the expression of potential lncRNAs [126]. They may indirectly affect lncRNA expression levels by modulating specific signaling pathways and biological mechanisms [126]. For instance, certain SNPs could regulate transcription factor binding sites, affecting the transcriptional activity of lncRNA genes, or interfere with lncRNA stability and function during post-transcriptional modifications [126]. Therefore, exploring the relationship between SNPs and lncRNAs can not only help reveal potential biological mechanisms but also provide new insights into diagnosing and treating psychiatric disorders. Identifying SNPs that specifically regulate lncRNA expression could support early detection and personalized treatment approaches for these disorders.

Despite the robustness of approach, several limitations warrant consideration. The heterogeneity of psychiatric and neurodegenerative disorders challenges the generalizability of the findings. Currently, there is no standardized protocol for detecting and quantifying lncRNAs, leading to variability across studies and laboratories. Additionally, there is a complete lack of overlap in data among different research groups studying the same diseases, further complicating the landscape. This lack of standardization poses a significant barrier to the widespread clinical adoption of lncRNA biomarkers. Moreover, lncRNA expression can be affected by various factors, including drug treatment, demographics, disease stages, and comorbidities, which complicates the interpretation of results compared to more established biomarkers and may potentially lead to confounding outcomes. Although numerous studies have highlighted the diagnostic potential of lncRNAs across various diseases using ROC curve analysis, it is important to recognize that such analysis is only one aspect of biomarker evaluation. Relying solely on ROC curve results cannot definitively establish any specific lncRNA as a biomarker for a particular disease. While this methodology provides a theoretical foundation for clinical prediction, it cannot be immediately adopted as a clinical predictive tool. Addressing these research gaps through well-designed comparative studies is essential for integrating lncRNAs into clinical practice and realizing their potential to enhance diagnostic accuracy and patient care.

Future research should prioritize studies that directly compare the diagnostic performance of lncRNAs against other biomarkers and traditional clinical methods, evaluating diagnostic accuracy, cost-effectiveness, and clinical utility. It is essential to acknowledge that lncRNAs, as candidate biomarkers for certain diseases, require extensive evaluation using diverse assays across different populations before being validated as biomarkers. Furthermore, they should be monitored in longitudinal studies. Currently, none of the lncRNAs have been rigorously assessed for their diagnostic potential in neurodegenerative diseases or psychiatric disorders. While animal models are invaluable for mechanistic studies, they do not fully capture the complexities of human diseases. Future research should aim to validate these findings in larger, more diverse human cohorts and explore lncRNA-targeted interventions in clinical trials.

## 5. Conclusions

LncRNAs play an important role in various physiological and biochemical functions of organisms, and intensive studies of lncRNAs have revealed their roles in the modulation of key response factors and regulators involved in epigenetic processes. As technology continues to be updated, researchers have identified lncRNAs as promising biomarkers for psychiatric disorders and neurodegenerative disorders. However, the current research on psychiatric disorders in which lncRNAs are directly involved is still insufficient. Future research efforts should focus on quantifying lncRNAs expression levels in the brain, identifying new potential lncRNA candidates, and elucidating their potential mechanisms in psychiatric disorders. These efforts aim to increase the potential of lncRNAs as biomarkers and to facilitate the development of innovative interventions for the treatment of psychiatric disorders.

## Figures and Tables

**Table 1:** Studies examining lncRNAs and psychiatric disorders.

Illnesses	Sample	Method	Main Finding	References
SCZ	39 patients and 51 CON	RT–qPCR	NONHSAG004550 ↑, NONHSAT125420 ↑	[26]
SCZ	138 patients and 63 CON	RT–qPCR	TCONS_00019174 ↓, ENST00000566208 ↓, NONHSAG045500 ↓, ENST00000517573 ↓, NONHSAT034045 ↓ and NONHSAT142707 ↓	[27]
SCZ	9 CON and 9 patients	RT–qPCR	MIAT ↓	[28]
SCZ	50 CON and 50 patients	Real-Time PCR	FAS-AS 1 ↓, PVT 1 ↓, TUG 1 ↓	[29]
SCZ	50 patients and 50 HCs	Real-Time PCR.	H19 in male cases ↑	[30]
SCZ	86 patients and 44 CON	Real-Time PCR.	PINT ↓, GAS5 ↓	[30]
MDD	60 samples	qRT–PCR	Linc00473 in female patients ↓	[31]
BD	50 patients and 50 HCs	qPCR	NKILA and COX2 expression in patients ↓	[32]
BD	50 patients and 50 HCs	Real-time PCR.	The expression of RMRP and CTC-487M23.5 in individuals ↑, RMRP ↑ and CTC-487M23.5 and DGCR5 in female patients ↓	[33]
BD	50 patients and 50 HCs	Real-time PCR	Expression levels of lincRNA-p21, lincRNA-ROR and lincRNA-PINT in patients ↓	[34]
BD	50 patients and 50 HCs	Real-time PCR.	expression in DILC and DICER1-AS1 in female patients ↑.	[35]

Note: ↓ or ↑: LncRNA expression levels were up-regulated or down-regulated. Abbreviations: SCZ: schizophrenia, MDD: major depressive disorder, and BD: bipolar disorder.

**Table 2:** Studies between lncRNAs and neurodegenerative diseases.

Illnesses	Sample	Method	Main Finding	Reference
AD	5 patients and 5 HCs	Northern blot	ACE1-AS Regulation BACE1 ↑	[75]
AD	25 mice	CRISPR-Cas9	Silencing of MAPT-AS1 increases neuronal tau	[76]
AD	9 patients and 8 HCs	RNA-seq	RP3-522J7, CCDC13-AS1, miR3180-2, miR3180-3, and CTA-929C8 ↑	[77]
PD	83 patients and 50 HCS	qPCR	LINC01783 ↑	[78]
PD	5 patients and 5HCs	Microarray Analysis	AC131056.3-001, HOTAIRM1, lnc-MOK-6:1 and RF01976.1-201 in circulating leukocytes patients ↑	[79]
PD	56 model mice and 8 HCs mice	RT–qPCR	GAS5 in SH-SY5Y ↑	[80]

Note: ↑: LncRNA expression levels were up-regulated. Abbreviations: AD: Alzheimer’s disease, PD: Parkinson’s disease.

**Table 3:** Candidate LncRNA Biomarkers for major psychiatric disorders, AD, and PD.

Name of lncRNA	Disease	Method	Main Finding	Reference
NEAT1	AD	Real-Time PCR.	NEAT1 ↑ in mice	[117]
	PD		NEAT1 ↑ can protect dopamine	[118]
	SCZ		NEAT1 ↓ in patients	[119]
MALAT1	AD	RT–qPCR	MALAT1 ↑ in PD in SH-SY5Y	[120]
	PD		MALAT1 ↓ in AD rats	
SNHG1	AD	RT–qPCR	SNHG1 ↑ in AD patients	[121]
	PD		SNHG1 ↑ in PD mice	[122]
H19	BD	RT–qPCR	H19 ↓ in BD patients	[123]
	PD		H19 ↓ in PD patients	[113]
	AD		H19 ↑ in AD mice	[124]
MEG3	AD	RT–qPCR	MEG3 ↑ in AD patients	[125]
	BD		MEG3 ↓ in BD patients	[123]
	SCZ		MEG3 ↑ in SCZ patients	[30]

Note: ↓ or ↑: LncRNA expression levels were up-regulated or down-regulated. Abbreviations: SCZ: schizophrenia, BD: bipolar disorder, AD: Alzheimer’s disease, PD: Parkinson’s disease.

## Abbreviations

ICD	International Classification of diseases
DSM	Diagnostic and Statistical Manual of Mental Disorders
MDD	major depressive disorder
BD	bipolar disorder
SCZ	schizophrenia
PD	Parkinson’s disease
BD	bipolar disorder
lncRNAs	long non-coding RNAs;
ncRNAs	non-coding RNAs
GABA	γ-aminobutyric acid
DCGR5	DiGeorge Syndrome Critical Region Gene 5
PBMC	peripheral blood mononuclear cells
SNPs	single-nucleotide polymorphisms
BDNF	Brain-derived neurotrophic factor
CUMS	chronic unpredictable mild stress
SERT	serotonin transporter protein
LOAD	late-onset AD
Aβ	amyloid β peptide
MPTP	1-methyl-4-phenyl-1, 2, 3, 6-tetrahydropyridine
MPP	membrane-permeable peptide
GWAS	genome-wide association studies
Crybb1	crystallin Beta B1
RT–qPCR	Real-time PCR;
RIP	RNA Binding Protein Immunoprecipitation Assay
PRISMA	Preferred Reporting Items for Systematic Reviews and Meta-Analyses
DR	dopamine receptor
ROC	Receiver Operating Characteristic
